# Patient Perspectives on Enhancing Clinician Communication About Pain in Sickle Cell Disease

**DOI:** 10.21203/rs.3.rs-7503185/v1

**Published:** 2025-09-08

**Authors:** Miranda Ravicz Adelmann, Michelle S Diop, Sharl S Azar, Dima Hendricks, Piper K Tingleaf, Miriam A Osei, Stephanie B Kiser, Ana-Maria Vranceanu, Areej El-Jawahri, Christine S Ritchie

**Affiliations:** Massachusetts General Hospital; Massachusetts General Hospital; Massachusetts General Hospital; University of Oklahoma Health Sciences Center; University of Oklahoma Health Sciences Center; Dana-Farber Cancer Institute; Massachusetts General Hospital; Massachusetts General Hospital; Massachusetts General Hospital; Massachusetts General Hospital

## Abstract

**Introduction:**

Pain in sickle cell disease (SCD) causes profound emotional and psychological consequences. Poor communication between clinicians and people with SCD can worsen the acute pain experience, yet strategies to improve these interactions remain unclear. We aimed to understand how people with SCD communicate with clinicians about pain and how patient-clinician communication could be improved.

**Methods:**

We conducted semi-structured qualitative interviews with people with SCD aged 16 and older (n = 30). We used rapid qualitative analysis to provide early insights into intervention development.

**Results:**

Five themes emerged: 1) people with SCD want clinicians to believe their pain experiences, to show they care, and to demonstrate knowledge about SCD; 2) SCD-related pain directly impacts the ability to communicate; 3) communication about SCD pain should be tailored in content and pace based on the pain severity; 4) prior experiences influence how people with SCD communicate about pain; and 5) healthcare system and institutional factors can positively influence patient-clinician communication in SCD.

**Discussion:**

Empathic communication can have significant impact on the pain experience of people with SCD. Training clinicians in SCD-specific empathic communication skills may improve pain care for this population.

## Introduction

People with sickle cell disease (SCD), a genetic condition affecting eight million individuals worldwide (GBD 2021 Sickle Cell Disease Collaborators, 2023), experience episodes of severe acute pain that often begin in early childhood (Kavanagh et al., 2022). Acute pain in SCD is caused by vaso-occlusive episodes (VOE), or vaso-occlusive “crises” (Savitt et al., 2014), in which abnormally shaped red blood cells obstruct blood vessels and prevent oxygen delivery to tissues. People with SCD are at risk for life-threatening complications including sepsis, stroke, and acute chest syndrome. The life expectancy in the United States is 52.6 years—more than two decades below the national average (Jiao et al., 2023). Severe VOE is treated urgently in the Emergency Department (ED), often with intravenous opioids. More than half of adults with SCD also have chronic pain from repeated tissue damage (Osunkwo et al., 2020; Smith et al., 2008), many of whom receive chronic opioid therapy (Han et al., 2016) and lack access to nonpharmacological pain care (Osunkwo et al., 2021).

Pain in SCD is associated with reduced health-related quality of life (Childerhose et al., 2023; Dampier et al., 2011; McClish et al., 2024), worse emotional wellbeing (Osunkwo et al., 2021), and higher rates of depression (Harris et al., 2023; Reader et al., 2020). The challenges of living with this life-threatening diagnosis and fear of death during VOE may also worsen the biopsychosocial pain experience (Booker et al., 2006; Gil, 1989; Oyedeji et al., 2025).

Patients often describe their experiences of acute pain management in the ED as contentious and distressing (Brown et al., 2015; Lattimer et al., 2010; Renedo et al., 2019). These experiences are linked to negative clinician attitudes and bias toward people with SCD related to their diagnosis, opioid use, and race (Freiermuth et al., 2014; Haywood et al., 2015). SCD is most common among people descended from sub-Saharan African, Middle Eastern, South Asian, Mediterranean, Latin American, or Caribbean regions, and people with SCD experience well-documented racial bias in health care (Guarino et al., 2024; Haywood et al., 2014; Maxwell et al., 1999; Miller et al., 2024).

Given persistent communication challenges between people with SCD and clinicians, interventions aimed at patient-clinician communication may improve patients’ pain experiences. However, it is not clear how to most effectively improve communication for this population. The Social Transactional Model for pain communication (Schiavenato & Craig, 2010), which has been previously applied to the SCD context (Collins et al., 2022; Schiavenato & Alvarez, 2013), identifies several potential intervention targets including patient factors, clinician factors, and the patient-clinician interface. There have been previous efforts to develop interventions to enhance communication between patients with SCD (Jean-Baptiste et al., 2022; Jenerette et al., 2014) and clinicians (Thomas & Cohn, 2006), but none have demonstrated changes in communication behaviors or patient-clinician interactions.

To develop effective interventions for improving pain communication in the acute medical setting, it is necessary to understand patient perspectives on the communication experience. We used qualitative methods to explore how people with SCD communicate with clinicians about pain and how patient-clinician communication could be improved.

## Methods

### Study Design and Ethics

We conducted semi-structured interviews with 30 people with SCD. Participants also completed a demographics survey via REDCap. A gift card for $50 was provided as compensation. The study was approved by the Institutional Review Board.

### Participants and Setting

Participants were recruited in person from the adult and pediatric sickle cell clinics at a tertiary hospital in the northeastern United States (“Clinic”). Eligibility criteria included age 16 years and older, SCD diagnosis (any genotype), and at least one lifetime hospitalization for SCD-related pain. Exclusion criteria included cognitive impairment impeding informed consent (per hematologist assessment), speaking a language other than English, or currently experiencing severe pain (per patient). Participants aged 16–17 years provided assent and a parent or guardian provided informed consent.

Purposive sampling was used to select participants with a range of ages and genders. Of 33 eligible patients who were approached, 30 completed the study and three withdrew due to competing commitments. Thematic saturation was reached after 22 interviews (Guest et al., 2006), but eight additional patients were recruited to facilitate caregiver recruitment for another study and all 30 patient interviews were included in analysis.

### Semi-Structured Interviews

We developed a semi-structured interview guide (Supplementary Material) to explore communication experiences using the Social Transactional Model of pain communication (Schiavenato & Craig, 2010). A think-aloud interview was conducted with a person with SCD to ensure appropriate language and content (DH), and the interview guide was revised to better situate pain within participants’ broader life context. We conducted interviews in person at the Clinic or by videoconference. All interviews were conducted by the primary author, a female physician who was unaffiliated with the Clinic, had no prior relationship with study participants, and had prior qualitative research training and clinical experience with SCD. For four interviews, a second researcher was also present. Observations were recorded immediately after each interview. Interviews were audio recorded, transcribed, and de-identified (mean length 46.1 min, range 23.5–65.0).

### Rapid Qualitative Analysis

We used thematic rapid qualitative analysis (RQA) to generate timely insights into potential communication interventions (Gale et al., 2019; Hamilton & Finley, 2019), aiming to complete qualitative data generation and analysis within six months (March to August 2025). To ensure rigor, we followed the PARRQA framework, a consensus-based checklist for RQA (Kowalski et al., 2024). Using interview recordings and transcripts, brief summaries were prepared by the primary author using a template aligned with the interview guide (Supplementary Material).

Summaries were transferred to a matrix and two authors collaboratively generated preliminary themes and subthemes which were cross-checked across interview domains and cases to ensure accuracy and completeness. The findings were presented to the Patient Advisory Council at our institution, a group of adults with SCD who receive care at the Clinic and meet monthly to discuss clinical issues and review research efforts. The members of the Patient Advisory Council confirmed that the themes accurately represented their lived experiences and made minor modifications.

## Results

### Participants

Thirty individuals with SCD participated in the study (Table 1). The average age was 27.1 years (range 16–52). One participant was < 18 years. Most participants were women (67%) and non-Hispanic Black or African American (63%).

Five themes were identified (Table 2): 1. people with SCD want clinicians to believe their pain experiences, to show they care, and to demonstrate knowledge about SCD; 2. SCD-related pain directly impacts the ability to communicate; 3. communication about SCD pain should be tailored in content and pace based on the pain severity; 4. prior experiences influence how people with SCD communicate about pain; and 5. healthcare system and institutional factors can positively influence patient-clinician communication in SCD.

### Theme 1: People with SCD want clinicians to believe their pain experiences, to show they care, and to demonstrate knowledge about SCD.

When asked how communication with clinicians could be improved, participants responded, “Believe us.” They identified barriers to recognition of their pain including having an “invisible” illness and “prejudices or just preconceived notions.” They spoke about the psychological harm of being repeatedly disbelieved or told that they “shouldn’t be having pain.” They appreciated when clinicians verbally affirmed their pain (“I hear you. I understand you”) and summarized their story to show understanding.

To assess clinicians’ “bedside manner,” participants used both verbal (“I hear you”) and nonverbal (“shifting his feet”) cues. Participants recognized that empathic clinicians alleviated their pain experience, whereas dismissive clinicians “kick[ed] you while you’re down.” Many participants highlighted that nurses provided comfort during their hospitalizations through “just simple acts [that] made me realize they really care.” Participants appreciated when clinicians cared about them as people and asked about their lives outside the hospital, rather than treating them as if they “happen to be the next patient.”

Participants shared more information with clinicians who cared about them: “If I feel like you’re just asking for the simple answer, then [I’ll] give you a simple answer.” They were more receptive to medical recommendations from clinicians who seemed to listen and care: “The biggest thing that helps is listening … ‘cause once that happens, then I can actually listen to what they have to say.”

However, effective communication did not rely on kind words alone. They described doctors who showed “fake kindness” and “never come back” to reassess their pain. To show they truly understood, clinicians should make a concrete treatment plan—and follow it. Participants also emphasized the importance of clinicians demonstrating knowledge of SCD. Several mentioned having been asked how long they had had SCD, which revealed poor understanding of this lifelong genetic illness. Communicating with clinicians who showed little knowledge of SCD increased stress for participants.

### Theme 2: SCD-related pain directly impacts the ability to communicate.

Participants described that speaking during VOE is impeded not only by pain (“if you’re a 10, you do not wanna talk”) but also by weakness, fatigue, and dyspnea from the VOE and by drowsiness from pain medications.

Severe VOE pain also hindered patients’ communication through extreme stress (“when I get stressed, it also affects my pain bad”) and vulnerability (“it’s demoralizing and just gut-wrenching”). They felt their medical care depended on the clinician’s arbitrary judgment: “It’s like every time I have to go back to the hospital, I have to think about … who’s gonna understand me this time? Who’s gonna hear me this time?”

Existential fear also shaped their communication about pain. Several participants described the fear of death from clinician mismanagement that accompanied each ED visit, especially when clinicians seemed unfamiliar with SCD or unmotivated to provide expeditious treatment (“you’re literally trying to advocate for your life”). One participant described the “desperation” that she felt as she tried to communicate with clinicians who seemed not to understand the potentially life-threatening nature of VOE.

Participants shared that their body language during pain episodes could be misinterpreted by clinicians. In some cases their body language visibly demonstrated severe pain (“screaming and writhing and grunting”). However, the quality of their pain could vary during VOE, and their body language would vary correspondingly: “Sometimes if I’m having a crisis, I’ll be in pain but I’m not crying, and then other times I’m screaming, crying.” This variation in body language led to misunderstanding by clinicians.

Participants’ body language was also impacted by their pain coping strategies. These included distraction (“playing with my jewelry”), humor (“I make a lot of jokes”), acceptance (“this too shall pass”), relaxation (“gotta just focus on my breathing”), and suppression (“my face has become stone cold”). These coping strategies sometimes led to perceived misunderstanding by clinicians who interpreted these behaviors as indicative of milder pain.

Due to the numerous communication difficulties during VOE, participants spoke about the importance of having a family member or advocate to “be my voice for me.” Not only were family members helpful in describing the pain to clinicians, but also they could physically find clinicians in the ED to help if pain was inadequately treated: “Then I don’t have to yell and be in pain and feel like no one’s hearing me.” However, participants recognized that family members may not always be available, and that some patients may prefer not to share medical information with their families.

### Theme 3: Communication about SCD pain should be tailored in content and pace based on the pain severity.

When first presenting to the ED with severe acute pain, participants preferred that communication with clinicians be brief, focused, and aimed at starting treatment as rapidly as possible. They suggested a list of questions that clinicians should prioritize (Table 3).

Participants had varying opinions about whether the initial brief assessment should use the numeric rating scale (Hawker et al., 2011). Some participants found the numeric scale useful, especially when pain made talking difficult: “It’s practical for us … at those times where we are so desperate in pain that we just don’t want to do much talking.” Many felt the numeric scale should be supplemented with additional details: “[The pain scale] isn’t adequate enough language to describe what’s happening, but I still use it just to help people get a gauge.” Some participants warned that the numeric scale can be misleading (“when I’m saying a low number … I’m still in a lot of pain”).

Participants did want clinicians to assess for SCD complications (e.g., infection, acute chest syndrome) that might be driving the pain (“I don’t want you to jump straight to it being sickle cell”). However, they warned that vital signs could be misinterpreted by clinicians: “Even though my vitals are saying one thing, my body is saying a different story.”

Many participants gave ED clinicians specific guidance on their initial treatment plan, despite negative responses from some clinicians (“how dare the patient tell me how to do my job?”). As one participant explained: “This may be your first rodeo, but it’s not mine. I know what it is I need, and I know that with the tools that I’m giving you, I have a better shot of getting out of this.” However, some participants did not know their medication doses for severe VOE (“what I normally use is OTC [over the counter]”).

As the pain stabilized, participants wanted clinicians to invite their input in developing a pain management plan (“we can meet in the middle”). They emphasized that clinicians should remain attentive to the pain, as it could unexpectedly and rapidly worsen during the hospitalization.

When the pain improved, participants would request that clinicians lower the dose of pain medication in preparation for discharge (“that’s the easy part”). They appreciated when clinicians were flexible about the timing of discharge due to the unpredictability of pain.

Throughout all stages of VOE, participants emphasized the importance of cultivating trust, respect, and recognition of the patient as the expert in their body. As one participant summarized, “Respect people. … A lot of it comes down to that.”

### Theme 4: Prior experiences influence how people with SCD communicate about pain.

Participants adjusted their communication approaches based on prior experiences. Several participants aimed for a “calm, cool, collected” demeanor with clinicians because “if you were to show those emotions, then … you’re just another person yelling at them.” From prior experiences of having their pain underestimated or disbelieved, some participants learned to display their pain more visibly. As one participant explained: “I have found myself … avoiding being as mobile as I can be. … If I’m just getting up and walking around on my own, and I’m in pain level 13, they’re gonna think I’m lying.”

Participants learned how to navigate the healthcare system, including communicating directly with clinicians who are “in charge of the care plan,” or escalating to a supervisor if needed. They reflected that even with these strategies, they still sometimes felt powerless to advocate for the medical care they needed: “If you have the ability to communicate, the know-how, and you’re talking to all these people, and still that doesn’t move the needle … imagine other people who don’t have the background, who don’t feel comfortable making a fuss.”

Participants spoke about the lasting emotional and psychological impact of negative healthcare experiences. They shared that “the emotional humiliation that you have to endure because of pain you didn’t ask for, it doesn’t go away.” Another participant spoke about the “gap of pain suffered” when appropriate treatment is delayed or inadequate, and how this suffering has an enduring effect.

Parents influenced participants’ communication styles in nuanced ways. Participants reflected on transitioning from childhood, in which their parents “did most of the talking,” to adulthood, in which “I was able to communicate on my own, because I knew exactly what I was feeling.” Several participants modeled their communication on their parents, especially regarding self-advocacy (“my mom raised me to be vocal”) and asking questions (“what would [my mom] ask?”). Some participants’ perspectives had diverged from their parents’, particularly around opioids for SCD-related pain. One participant spoke about how her parents encouraged her to report lower pain ratings for faster hospital discharge.

Several participants spoke about how bias and stigma related to race, opioid use, and SCD diagnosis impacted how they communicate with clinicians. They tried to avoid language that would invoke clinician suspicions about opioid-seeking behavior: “I’m trying not to say that I need opioids because that stigma is always in the back of my head that I’m just a drug seeker.” They spoke about the influence of their race on clinicians’ perceptions: “I was a young girl versus many Black boys who grow up, and they’re six feet at 12 [years old], right? So you make yourself smaller. You make your voice lighter. You say, ‘Please, can I? I’m in a lotta pain.’” As one participant summarized, “They made sickle cell such a dirty illness. They make it more about the drugs and seeking drugs than anything.”

### Theme 5: Healthcare system and institutional factors can positively influence patient-clinician communication in SCD.

Participants described differences in communication experiences across medical settings (ED, inpatient, clinic, and infusion). They noted that communication tended to be more positive in pediatric settings and more challenging in the ED. However, despite communication challenges, they also described positive experiences across all medical settings. Some had noticed recent improvements in their ED experiences: “The ED was 1000% prepared for me. … I’m just so thankful and grateful for it.”

Several participants noted increased awareness of SCD among clinicians compared to prior: “There are some cases here and there where you have some people who aren’t educated on the illness. I feel that’s more rare nowadays.” In the hospital, participants noticed that “folks were coming by to check in and see how I was doing,” which they attributed to “the word is going around.”

Participants spoke about the impact of having a trusted multidisciplinary Clinic team. They noted that communication felt easier with clinicians who knew their personal and medical history, and who advocated for them with ED and inpatient clinicians. One participant explained: “You can advocate for yourself, but it’s even better if you have a team that understands your illness in and out.” They also felt empowered by the Clinic’s local and national advocacy efforts: “You root for that person that comes along and fights your battles.”

Participants noticed the impact of two large-scale interventions that have been widely adopted for SCD: publishing a personalized treatment plan for VOE in the patient’s medical record (“acute care plan”) and accessing the infusion center (“day hospital”) as an alternative to the ED for acute pain management. Several participants noticed that ED pain management was smoother with an acute care plan, although they noted that some clinicians did not follow the acute care plan while others adhered too rigidly. In the infusion center, most participants reported excellent communication and felt these interactions could be a model for SCD pain care in other settings like the ED.

## Discussion

We used semi-structured interviews to understand the perspectives of people with SCD on improving pain communication with clinicians. We generated five themes that synthesize participants’ communication experiences and suggestions for improvement.

Participants emphasized that clinicians should express empathy for their pain and validate their pain experiences. Validation involves verbalizing that the person’s experience is “understandable and legitimate” (Edmond & Keefe, 2015; Linehan, 1997) and “worthy of social acceptance and support” (Nicola et al., 2022). For people with SCD pain, clinician communication has the power to either alleviate suffering from the VOE or compound the trauma of these severe pain episodes.

Participants described the specific verbal and nonverbal cues they used to assess clinicians’ attitudes. They observed clinicians’ body language, facial expressions, and tone. They appreciated clinicians making affirmative statements (“I hear you”) and summarizing their story to confirm understanding. Importantly, they noted that bedside manner must be paired with action, including reliable updates and following through on the plans discussed. Prior research has described that people with SCD feel that clinicians disregard their pain (Adegbola et al., 2012; Booker et al., 2006; Coleman et al., 2016; Collins et al., 2022). Our study builds on this prior work by demonstrating behaviors that clinicians can use to convey empathy to patients with SCD.

We also delved into the specific strategies that people with SCD use to communicate with clinicians about pain. To expedite pain treatment, participants provide information on the medications that work best, but remain cognizant that clinicians might misinterpret this guidance as opioid-seeking. To build rapport with clinicians, participants maintain a calm demeanor, but know that this composure could lead clinicians to underestimate their pain. Overall, participants described a process of pain communication in which they adjust their own communication style to access proper medical care—an exhausting burden for people seeking urgent treatment. Prior research has described that negative healthcare experiences shape communication in SCD (Ciribassi & Patil, 2016; Young et al., 2020). Our study describes how people with SCD learn and implement these specific communication strategies.

There have been some prior efforts to develop interventions for patient-clinician communication in SCD. A patient-focused intervention aimed at improving assertive communication paradoxically resulted in increased awareness of stigma (Jenerette et al., 2014). A pilot intervention aimed at improving patients’ skills in providing relevant information to clinicians showed initial acceptability (Jean-Baptiste et al., 2022). Clinician-targeted interventions have included a pilot study to reduce implicit bias among clinicians (Mulchan et al., 2024) and a communication training that demonstrated increased confidence in communicating with patients with SCD but did not examine clinician behaviors or patient-clinician interactions (Thomas & Cohn, 2006). Future research should investigate how clinician communication skills trainings might improve pain communication with people with SCD.

An intervention aimed at improving patient-clinician pain communication must integrate the broader sociocultural and healthcare context (Schiavenato & Craig, 2010). Poor communication with people with SCD is linked to systemic bias related to race, opioid use, and SCD diagnosis. Participants described little accountability for delayed or inadequate pain care, leaving them vulnerable to whether individual clinicians recognized the urgency of VOE. Effective communication depends on well-trained clinicians assessing patients at the appropriate time and acknowledging the historical biases that have shaped current SCD care.

These findings may help improve communication not only in the ED but also for SCD pain care in other settings. Cognitive-behavioral approaches have shown benefit for some patients (Anie & Green, 2015; Jonassaint et al., 2024; Palermo et al., 2024) and more studies are needed to examine psychological interventions in SCD. Delivery of pain coping interventions may be more effective if clinician communication is tailored for people with SCD.

This study also highlight participants’ stories of positive clinician interactions, particularly with nurses, that contrast with the predominantly contentious interactions described in prior research (Alleyne & Thomas, 1994; Childerhose et al., 2024; Ciribassi & Patil, 2016). Our data offer an important balance to the literature and demonstrate that caring and knowledgeable clinicians can meaningfully impact SCD care. Moreover, implementation of existing SCD interventions including acute care plans (Siewny et al., 2024) and a day hospital (Lanzkron et al., 2021) at our institution have subjectively benefited our participants. These findings suggest that interventions to improve care for people with SCD can have major impact.

Our study has several limitations. The generalizability of our findings is limited by recruitment from a single institution. The Clinic at our institution has made substantial efforts to improve medical care for patients with SCD, and our participants’ experiences may be more positive than those at other institutions. Also, we excluded potential participants who spoke a language other than English, whose perspectives on healthcare communication should be explored in future studies.

In conclusion, we conducted in-depth qualitative interviews with people with SCD to explore perspectives on how communication with clinicians could be improved. Participants emphasized that to improve communication, clinicians should show they care about the patient as a person and believe their pain experience. Future directions may include communication skill building for clinicians to enhance empathic communication and pain validation.

## Supplementary Files

This is a list of supplementary files associated with this preprint. Click to download.

- JCPMSSCDcommunicationSupplementaryMaterial.docx

## Figures and Tables

**Table 1:** Participant demographics (n = 30).

Demographic	Mean (SD) or n (%)
Age (years)	27.1 (8.7)
Sex/Gender	
Female/Woman	20 (67%)
Male/Man	10 (33%)
Race/Ethnicity	
Hispanic/Latino	9 (30%)
Non-Hispanic Black/African American	19 (63%)
Other	1 (3%)
Prefer not to respond	1 (3%)
Genotype	
HbSS	23 (77%)
HbSC	7 (23%)
Employment	
Student	11 (37%)
Full-time work	8 (27%)
Part-time work	2 (7%)
Not currently employed and receiving disability benefits	2 (7%)
Not currently employed and not receiving disability benefits	7 (23%)
Relationship	
Single	17 (57%)
Married	5 (17%)
Living with partner	2 (7%)
In a relationship	4 (13%)
Divorced	1 (3%)
Prefer not to respond	1 (3%)

**Table 2:** Themes, subthemes, and key quotes generated from rapid qualitative analysis. Participant sex and age are indicated by male (M), female (F), and age group (years).

Themes	Sub-Themes	Key Quotes
*Theme 1: People with SCD want clinicians to believe their pain experiences, to show they care, and to demonstrate knowledge about SCD.*	Patients want clinicians to show that they believe the patient’s pain experience.	“I think it starts with, **you have to believe me.** If you don’t even believe me, how can you treat me?” (24–27F) “There’s been instances where just people don’t take you seriously if they can’t see. ‘Cause **it’s an invisible illness**, so if they can’t see how much pain you’re in, they don’t believe it or they don’t care.” (24–27F) “I could cry. I could be short of breath, I could literally just be fading in front of your eyes but **because of prejudices or just preconceived notions or ideologies, a lot of people suffer**. It’s really heartbreaking.” (24–27F) “They would say, ‘There’s nothing on your MRI, we don’t know why you’re in pain and **you shouldn’t be having pain**.’ It was always like, ‘you shouldn’t be, you shouldn’t be, you shouldn’t be’ despite me experiencing pain.” (24–27F) “Listen. Listen to your patients**. Listen your patients, and talk to them. Don’t discard them.** Just listen to what we’re telling you ‘cause the average sickle cell patient just wants a normal life. We want to be normal, just like you, just like them. We want to go to school. We want to go to college. We want to get a degree. We want to have a job. We want to get married. We want to succeed in life. We don’t want this life. We don’t want to fail in life because we have an illness.” (40–52M)
	Patients use verbal and nonverbal cues to assess clinicians’ attitudes.	“That last doctor … it was as if you could possibly **see the smoke coming out of his ears.** He was just really annoyed to the point where he’s like, ‘Well, if you don’t like it, you can always just go home.’ … You could just tell he was rigid. He was upset. He was shifting his feet, just not good body language.” (24–27F) “I just need to know that they’re actually being attentive. That’s the only thing that I look for if a doctor’s talking to me. I just need to know that you’re not about to try to rush out of the room to your next patient. **That’s the body language that I’m looking for.** Are you going to sit down and actually be open to having the conversation, or are you going to stand right at the door to just be in and out?” (20–23F) “I like to hear the same words. **‘I hear you. I understand you.’”** (20–23F) “When they **repeat what I’ve said.** They acknowledge what has been said or what has been done. … Them repeating what I’ve said or repeating my medical history and my treatment plan helps create that trust.” (36–39F)
	Caring behavior from clinicians alleviates the pain experience, and unkind demeanor worsens the pain. Many nurses provide compassionate care.	“Bedside manner is very important. **Bedside manner will take folks a long way.** I’ve had moments where, wow. That nurse or that doctor is in the field because they love it…. I’ve had other nurses or professionals, [and] the bedside manner, it’s just really bad. Imagine you’re already vulnerable, and **someone is just kicking you while you’re down.** It’s the worst feeling.” (36–39F) “The whole experience also impacts my pain because we know that there’s **a whole feedback loop with the stress** that we go through and the pain that we feel, so actually showing some empathy is part of healthcare. It’s part of you not making me worse by inducing more stress in me.” (28–31F) “If they realized that I was just really down in the dumps, [the nurses] would just go out of their way to make me feel better. **Just simple acts** … made me realize they really care.” (24–27F) “**I loved every nurse** that I have. … I felt like I had a big support group of nurses. My nurses were my more support team ‘cause they were front row.” (16–19F)
	Patients appreciate when clinicians care about them as people and understand the pain in the context of their lives.	“Are you helping me because you want to help me …? Or is it because **I happen to be the next patient?”** (32–35M) “I feel what really impacts the way you communicate to your providers and stuff is the way they approach you and the way **they try to form that bond with you** … They sit and talk to me and they ask me how school’s going and they interact with me. They’re not just there to like, ‘Oh, I’m here to give you your IV fluids and see you later.’“ (20–23F) “I think it’s just understanding where people are in **their journey in life.** ‘Cause that would really inform how you approach some of this—some of these things.” (36–39F) “Just because **work, life, family, money**—it all plays a factor to it. Unfortunately, sickle cell touches it all.” (36–39F)
	Patients share more information with clinicians who seem to believe and care about them.	“If I feel like you’re just asking for the simple answer, then [I’ll] give you a simple answer and **not tell you what’s actually wrong** or how bad it’s hurting.” (32–35M) The biggest thing that helps is listening, getting the doctors to listen to you. ‘Cause once that happens, **then I can actually listen to what they have to say** on what I need.” (24–27F) “‘I’m here to help you feel comfortable. This is tough. We’re gonna get through it together.’ That’s building rapport to me. That trumps everything whatsoever. I feel like, yeah, we’re in this together. **Then you can start to ask me questions.”** (36–39F)
	Authentically caring about patients involves communicating a concrete plan and following through.	**“Actions speak louder than words.** Being heard is nice but I want you to actually do the right thing.” (24–27F) “[Doctors will] give you that, ‘Yeah, okay. Well, I could see what I could do,’ and **never come back.”** (20–23F) “‘Cause sometimes they’ll act as if they understand. Then they’ll go and they’ll be like, ‘Okay. We’re giving you the pain medication you asked for, but we’re only gonna give you half of it.’ **Sometimes, some doctors just have fake kindness.”** (24–27F)
	Patients prefer to communicate with clinicians who seem knowledgeable about SCD, including recognizing VOE as a medical emergency.	“I have to explain what sickle cell is—and yet **they’re the ones who are supposed to take care of me.”** (24–27F) “It’s just—it’s even scary getting in the ambulance with the EMT and her literally asking me the question, **‘So, how long have you had sickle cell?’** Not even knowing it’s something you’re diagnosed with since birth.” (24–27F)
	Clinicians and others cannot fully understand the pain experience.	“I feel like unless you live it, **no one can honestly ever understand** it to a complete extent.” (20–23F) “Sometimes, even though I feel like I have enough language to describe it—I don’t know—**there’s always a void of understanding.”** (20–23M) “It’s me by myself that is feeling exactly what I am feeling and trying to communicate that to those who don’t understand or make it a point to not understand. **It’s exhausting. …** It would be nice if they just got it.” (24–27F)
*Theme 2: SCD-related pain directly impacts the ability to communicate.*	Pain, shortness of breath, fatigue, and weakness from VOE hinder communication.	“Cause sometimes you’re in so much pain, you don’t wanna talk. You just give a number. **If you’re a 10, you do not wanna talk.”** (40–52M) “Half the time I’m in so much pain, **I can’t breathe.** The other half, I’m in so much pain, that it keeps you awake, and every time they give me something for relief, I just go right to sleep ‘cause I’m so tired from staying awake so long from the pain.” (40–52F) “Usually the first sign I’m getting better is **I’m sitting up and talking** a lot more rather than lying down and sleeping.” (24–27F)
	Pain medications can cause drowsiness that hinders communication.	“‘Cause obviously, the **pain medicine makes me sleepy.** Then it’s like I am easier to be annoyed.” (20–23M) “Or I’m asking questions. You’re irritated. I’m not trying to be difficult. **I’m also drowsy, and I just want to get clear understanding**, ‘Why do I need this extra medicine?’“ (36–39F)
	Stress and vulnerability of the pain experience impact communication.	**“Because when I get stressed, it also affects my pain bad. Bad, bad, bad.** I’ve tried to change that a little bit because I just get worse. When I cry or when I hyperventilate or when I’m arguing with someone or trying to advocate for myself, it just gets worse.” (20–23F) “When your body is under that amount of stress resting is hard because **pain is as much physical as it is mental.** I couldn’t shut my brain. I couldn’t sleep. I would try and when I would finally get a moment of rest, it would be like 15 minutes and then I’d wake up again because the pain was so bad. It was unbearable.” (24–27F) “I hear you, but I also can’t hear you. I was aware, I was coherent, but **truthfully my mind was elsewhere because I was in so much pain.** I was, I guess, dissociating a lot ‘cause I was in so much pain.” (24–27F) “It’s like every time I have to go back to the hospital, I have to think about ‘Okay, what’s gonna happen now? What’s gonna be said now? What’s gonna be said this time? Who’s gonna understand me this time? **Who’s gonna hear me this time?’”** (36–39F) “I knew that I was in good hands even when my family wasn’t here, which was a relief ‘cause there’s nothing like being **in such a vulnerable state** and where your family can’t be with you and you’re in the hands of people who are a bit careless. **It’s demoralizing and just gut-wrenching.”** (24–27F)
	Patient’s communication is shaped by fear of death from mismanagement.	**“You’re literally trying to advocate for your life**, for yourself. Especially, a lot of the times, I’m alone in the ER … I feel hopeless. Like, ‘You’re not listening to me. You’re not understanding me. This is my life. It’s not a joke. At least try to put yourself in my shoes.’” (20–23F) “Now, I don’t argue anymore. I really used to argue, and they look at you like you’re crazy because you’re in this **state of desperation.”** (20–23F) “I have my cousin who passed away at 30. I think also a lack of resources. **Same illness, same everything.”** (36–39F) “The reality of my life hits me every single day. Which is I’m a woman that’s chronically ill. **My illness is fatal.”** (24–27F)
	Body language can be misinterpreted by clinicians, especially when patients are using pain coping strategies.	“I have different types. Sometimes if I’m having a crisis, I’ll be in pain but **I’m not crying, and then other times I’m screaming,** crying but I feel it’s still really bad pain. It’s just hard to explain, yeah, I’m not crying, but this is still really painful.” (16–19F) “Growing up, **my face has become stone cold**, and it would be hard for other people to discern how much pain I’m in without me verbally telling them.” (20–23M) “I make a lot of jokes, but because **it’s the way I cope.** Because if I don’t laugh, then— yeah, very hopeless.” (20–23F) “‘Cause being able to breathe through the pain and—one of my phrases I like to say is, ‘This too shall pass.’ This is something that’s just happening at the moment. It’s not the state that I’m gonna be in forever. **I just gotta just focus on my breathing** and go through the pain versus deal with it.” (36–39M) “A lotta times, I will—**to distract myself, will be playing with my jewelry.** I wear rings and things like that, so I’ll either play with those or my necklace.” (20–23F) “If it’s impacting me that bad, my facial expressions will be an indication. … It’s like, ‘Hey, this person is **screaming and writhing and grunting.”** (16–19F)
	Family members or caregivers facilitate communication with clinicians, but are not always available.	**“I feel like a horror movie.** You’re in so much pain, and you’re yelling out, and no one’s listening to you, and no one hears you. … Having someone there to advocate for you is **night and day.** Makes things go faster ‘cause then I don’t have to yell and be in pain and feel like no one’s hearing me.” (28–31F) “If I go to the ED 9 times out of 10, when I am in a lot of pain, I can’t talk because it’s too much. My sister or my brother or mom or dad, whoever’s with me, friend or a cousin, they’ll step in and definitely **be my voice for me.”** (24–27F) “What works well is always having family around and available and them staying updated, but I know that’s up to me. **Not every patient wants their family updated** and knowing what’s happening with their health.” (36–39F)
*Theme 3: Communication about SCD pain should be tailored in content and pace based on the pain severity.*	*Early VOE (very severe pain)*: Communication is focused and action-oriented.	“When you’re in pain, you don’t wanna talk too much. When you’re in pain, you don’t want to have a hundred questionnaires like, ‘What’s your eye color?’ **Give me the meds and then we’ll talk later.”** (36–39F) “‘I want to help you get comfortable in the next 30 minutes. Just let me know where the pain is so I can giveyou something that will **give you some reliefbefore we start to talk.”’** (36–39F) “Asking too many questions, but not necessarily the right questions. You can ask me a lot of questions **as long as they’re the right questions.”** (36–39F)
	Numeric pain scale can be useful for rapid pain assessment, but can also be incomplete or misleading.	“I love that they always ask, ‘What number?’ like, ‘What’s your level of pain?’ **I think that’s a great start.”** (36–39F) “[Pain scale] isn’t adequate enough language to describe what’s happening, but **I still use it just to help people get a gauge.”** (20–23M) “I think it’s practical for us because it helps us put a number to our pain, and at those times where we are so desperate in pain that **we just don’t want to do much talking.”** (40–52M) “The scale usually works, but if they give me a scale 1 to 10, and I say a 12, and you’re not responding like a 12, **then there’s no point in you asking me** because you’re not actually listening.” (40–52F) “I think it’s different for someone who just came into the ER from a car crash or an injury than someone who’s dealing with pain from sickle cell because **it’s something that we were born with.** What may feel like a 10 out of 10 for someone else, it may feel like a five out of 10 to us. When I am saying a high number, **I mean that I could literally drop dead**, and when I’m saying a low number, I still mean that I’m still in a lot of pain.” (20–23F)
	Perspectives differ on the usefulness of describing the quality of pain.	“I actually think **describing the pain is probably the least impactful** to [clinicians] and to the outcome of my care, so I don’t, which is, I guess, reflective of our medical system.” (28–31F) “[My mom] was really the one talking about **you have to be descriptive** so that they can try to see where you’re coming from. Be very detailed.” (24–27M)
	Patients do want clinicians to assess for serious complications of SCD. Vital signs can be misleading.	**“I don’t want you to jump straight to it being sickle cell** and you wanting to just treat me with meds.” (36–39F) “I try to let them know that even though my vitals are saying one thing, my body is saying a different story. Because **your vitals cannot measure what the story is**, then you’re not getting an accurate full picture, and it’s the full picture you need to be able to help me out of this crisis.” (40–52F)
	Patients share their medical history to guide appropriate treatment (sometimes with negative response from clinicians). Some patients are less familiar with specific medication regimens.	“I don’t have to be combative, but I can also be assertive and let them know that **this may be your first rodeo, but it’s not mine.** I know what it is I need, and I know that with the tools that I’m giving you, I have a better shot of getting out of this.” (40–52F) “No matter how much you try to say that or advocate that for yourself, they won’t understand that that’s not enough medicine for you. Even though you know your pain regimen, and **you know what works for you because I do this all the time.** I’m admitted all the time.” (20–23F) “Not that every doctor has an ego, but you’re the doctor for a reason. **How dare the patient** tell me how to do my job.” (40–52M) “You can ask me what I normally use, but I think that changes in the hospital setting. **Because what I normally use is OTC** [over the counter], right?” (36–39F)
	*Middle VOE (moderate pain).* Communication should be collaborative.	“I struggle with people who are just coming and telling me what’s happening. … That’s why I feel like so much of this would be fixed with just simple conversation. I’m not asking to always have things my way, but I’m saying that **we can meet in the middle somewhere.”** (32–35M) “If I didn’t really wanna make a change that day, they’d be like, ‘Okay, we don’t have to make a big change but we can make a small change and if it doesn’t help too much, then we can always go back.’ It was a lot of, **‘Let’s at least give it a try** and if it doesn’t work, then we can reevaluate.’ … If something needed to be changed, they had no problem shifting the way things were going. **They made me comfortable”** (32–35M)
	Clinicians should be attentive to changes in pain during the hospitalization.	**“Sickle cell pain can be temperamental** and if you leave it alone for too long, a seven can go right back to a 10. Being at a senven for an hour doesn’t mean the pain is getting better, just that it’s currently under control.” (24–27F) “They’re asking me what hurts. I’m like, ‘No, I’m here for sickle cell. That’s why I’m crying so much. **I’m having a relapse right now in my crisis.’”** (24–27F)
	*Late VOE (improvedpain)* Patients appreciate flexibility and agency in weaning pain medications.	“‘I feel like I’m getting better. **Do you think you guys could probably try to transition me to orals** instead of being on the IV or something?’ I’d be trying to get out of the hospital as quick as possible.” (20–23F) “I just say that I would like to go on orals. **That’s the easy part.”** (20–23F) “‘Oh, you’re in control. **You tell us when you’re ready to switch.’** I like that, that they give you—they don’t rush you to get out of the hospital. … **I don’t want to leave to end up coming back** just the same night.” (20–23F)
	*AH stages of VOE*: Communication should demonstrate trust, respect, and recognition of the patient as the expert in their body.	“I just think **overall empathy and respect** no matter who it is, what the situation is or what your preconceived notions are … focus on the situation at hand and deal with it as best you can with that empathy and respect.” (40–52F) “I can hear you ‘cause you are an expert in medicine, but you have to also hear me **‘cause I’m an expert in my body.”** (36–39F) **“Respect people**… A lot of it comes down to that.” (32–35M) “When the nurses and doctor treats the patient as someone who’s on the team, essentially. They don’t look at the patient as just someone who’s sick and not feeling well, so let’s just do whatever we can think of. It’s about treating that patient as a team member and being like, **‘Okay, let’s hear them out, and then let’s expand.’** ‘Cause again, even though I’m not a professional, **I know my body the best.”** (20–23M)
*Theme 4: Prior experiences influence how people with SCD communicate about pain.*	Prior healthcare experiences make patients more cognizant of how they appear to clinicians	“If you come off too harsh or start yelling and things, that’s really not going to get you anywhere because they are dealing with people yelling at them all the time. … It is really difficult to do ‘cause you are upset. You are angry. If you were to show those emotions, then you’re not taken seriously, and **you’re just another person yelling at them.”** (20–23F) “I try to keep my composure together. … I try to keep it **calm, cool and collected.”** (24–27M) “Sometimes you have to—**not play it up, but you have to really express on your face how intense the pain is** ‘cause I think some people just by looking at me don’t realize that I’m in that much pain when I am in that much pain.” (24–27F) “I have found myself … avoiding being as mobile as I can be. … If they see I can’t walk on my own, they see I can’t move around, or if they see I can’t hold anything, or I **lift my arms or even crawl, then they’re more responsive** … whereas if I’m just getting up and walking around on my own, and I’m in pain level 13, they’re gonna think I’m lying.” (40–52F)
	Patients learn from experience how to navigate the healthcare system.	“Advocating with them helped me figure out how to talk to the nurses, not only how to talk to the nurses, but also **how to go up through the seniority** of the nurses to get the help that you needed.” (24–27F) “I try to find out who’s the one that’s in charge of the care plan and try to **talk to them directly.** Then if that doesn’t work, then that’s when I get my [Clinic] team to step in.” (36–39M) “I would say I have a lot of privilege. Just having an educational background that allows me to … put what I’m going through into the context that the medical providers will understand. … If you have the ability to communicate, the know-how, and you’re talking to all these people, **and still that doesn’t move the needle** … imagine other people who don’t have the background, who don’t feel comfortable making a fuss.” (28–31F)
	The pain and shame from being ignored and undertreated can have lasting consequences.	“Sickle cell pain can get treated, and after a few hours or a few days, it goes away, but the **emotional humiliation that you have to endure** because of pain you didn’t ask for, it doesn’t go away. It stays with you your whole life.” (40–52M) “They should have to justify not giving adequate care. I think people are too okay with like, ‘Oh, okay. Sorry you didn’t get adequate care before, but we’re moving on.’ **That gap of pain suffered**—you know what I mean? It’s not like cancer, where if they messed up and you didn’t get a week of chemo, you can measure the effect on the cancer. For us, they just chalk it up to suffering as if that’s gone, but it does have an impact.” (28–31F)
	As they transition from pediatric to adult SCD care, young adults learn through experience how to communicate with clinicians.	“When I was younger, my mom did most of the talking. As I got older, I was able to communicate on my own, because I knew **exactly what I was feeling.”** (36–39F) “I spent 10 hours in the waiting room in excruciating pain, and I was just crying and sobbing. I just didn’t know how to communicate my pain. That, honestly, did shape how I communicate now, because **I would never, in my life, do that ever again.”** (20–23F) “As I’ve gotten older, I just realize that if I’m gonna get healed, **I have to be as transparent as possible** and in the quickest way as possible as well so that I can get out of the hospital as fast as possible.” (20–23M)
	Parents influence how young adults communicate about pain.	**“My mom raised me to be vocal** and not be pushed around. Just be an advocate for myself if she wasn’t there to advocate for me.” (16–19F) “I feel like I can express myself more— kind of get into the thought of like, **what would [my mom] ask?** Questions she would probably say.” (20–23M) “For example, when I would go to the ER, **[my parents] told me to not say I was in so much pain because then I could come home sooner** or if I was admitted and I was at an eight, but then got down to a seven … feel free to come home. But a seven is terribly high for me. I just constantly had this negative feedback I guess about my disease and things I was doing wrong.” (24–27F)
	Prior experiences with bias/stigma shape patients’ communication styles.	“I was a young girl versus many Black boys who grow up, and they’re six feet at 12 [years old], right? So you make yourself smaller. You make your voice lighter. You say, ‘Please, can I? I’m in a lotta pain.’ You try to convey and humanize yourself as much as possible. I didn’t really start using those tactics until after 18 because then **I realized that I’m not automatically seen as a human anymore. …** You’re seen as a potential drug seeker. You’re seen as faker.” (28–31F) “I’m trying not to say that I need opioids because **that stigma is always in the back of my head** that I’m just a drug seeker.” (40–52F) “Especially sickle cell does happen predominantly to minority groups. … **Are they even going to listen to me** because I’m a Black woman?” (24–27F) “I personally hate having to ask for pain medication ‘cause I don’t wanna seem like I’m someone who relies on it. **I feel there’s that stigma** when it comes with patients like myself.” (36–39M) “Its almost like **they made sickle cell such a dirty illness.** They make it more about the drugs and seeking drugs than anything.” (40–52M)
*Theme 5: Healthcare system and institutional factors can positively influence patient-clinician communication in SCD.*	Many participants have had positive health care experiences across medical settings.	“The ED was 1000% prepared for me in an instant, and it was just **like night and day.** I don’t know who implemented that or when it started, but I’m just so thankful and grateful for it because I came in from < Hospital > in the **most traumatizing experience** I’ve ever had in a hospital in my life.” (40–52F) “Honestly, everybody was nice, **treated me nicely.** Everything went smoothly. At no point did I feel like I just build up tension or anything like that, I never felt that from my end.” (32–35M) “I had some **pretty great nurses and pretty great doctors.** … In terms of me communicating my needs and them being as clear as possible as to what they can do or as to what they’ll try and how patient they were, it was pretty awesome.” (24–27F)
	Quality of communication differs across care settings.	The nurses were nice; the doctors were nice. Peds [pediatrics] for sickle cell was beautiful. People take you seriously. You say, ‘I’m in pain.’ They say, ‘Let me help you.’ It’s a totally different land. Then you turn 18, and it’s almost like—you know those movies **where they start out in heaven, and then they kicked to Earth?** That’s exactly what it feels like.” (28–31F) “The emergency room, **it still needs some help,** and it’s come a long way. The hospital, to be honest with you, once I’m admitted, it’s pretty smooth sailing.” (40–52M)
	Participants have noticed increased awareness of SCD compared to prior.	“They’re seeing a lot more cases when it comes to sickle cell, so I feel they’re a lot more understanding. There are some cases here and there where you have some people who aren’t educated on the illness. I feel that’s **more rare nowadays.”** (36–39M) “I feel now sickle cell is being approached and **there’s more awareness** to it as to back in the day I feel there wasn’t as much. ‘Cause even my professors would be like, ‘What is sickle cell?’ I wouldn’t even know what to tell them.” (20–23F) “I had to grow up at a time where **you didn’t have a lot of people talking about sickle cell.** Those that had sickle cell around me didn’t want to talk about it. Basically, I would’ve talked more about it, be more open about it. It’s nothing that I need to be embarrassed about.” (40–52M) “I think, too, the **word is going around** because this was different from normal. I think more people are either more understanding to the illness or I don’t know, but folks were coming by to check in to see how I was doing.” (36–39F)
	Multidisciplinary Clinic team supports communication through continuity, expertise in sickle cell, and advocacy.	“Ever since Dr. <Name > came into play, he’s pretty much **changed a lot of the culture.”** (40–52M) “I wish Dr. <Name > could **teach classes on how to talk to sickle cell patients** because he just understands.” (20–23F) “When we see a doctor on the team fighting, going to congressmen and senators to better treatment for sickle cell specifically—I see Dr. <Name > on Twitter all the time, and I love watching him go toe-to-toe with people that are ignorant about sickle cell that still have that stereotype. **You root for that person that comes along and fights your battles.”** (40–52M) “You can advocate for yourself, but **it’s even better if you have a team that understands your illness** in and out, and what it takes to help you make it through the tough times.” (36–39M) “I’ve been goin’ to [hospital] since I was a child. … They know my life. I know, relatively, most of theirs. Its like a family type of environment, **like a safe space.** Talk about everything. Like I said, it feels like home.” (24–27M)
	Communication experiences have been improved by use of infusion center and acute care plans.	“The nurses [in the infusion center]— they’re so great. They’re so good. They’re so sweet. If that model could be taken from the infusion center, put in the ER—**I think ER would be changed forevermore.”** (36–39F) “Well, when they pull my name up, usually **all the meds I take when I’m having a crisis** are on the list and then they usually just follow that and then everything’s fine.” (20–23M)

**Table 3:** Patient perspectives on the important questions clinicians should ask when a patient with sickle cell disease presents with severe acute VOE.

Important Questions for Severe VOE
Where is the pain? When did it start? Is this sickle cell pain that you’ve experienced before, or is it different? How severe is the pain? If you’re familiar with using the pain scale, how would you rate your pain from 0 to 10? How has the pain impacted your activities, or your ability to move, breathe, focus, or speak? What have you tried already? Do you know what medications work best for you? What else brings comfort in addition to medications? Do you have someone with you? Should we call someone on the phone?

## Data Availability

Anonymized quotations are provided within the manuscript. Complete qualitative data are not publicly available to protect study participant privacy.
